# The Manufacturers’ Perspective on World Health Organization Prequalification of In Vitro Diagnostics

**DOI:** 10.1093/cid/cix719

**Published:** 2017-08-17

**Authors:** Sébastien Morin, Nelli Bazarova, Philippe Jacon, Stefano Vella

**Affiliations:** 1 HIV Programmes and Advocacy, International AIDS Society, Geneva, Switzerland; 2 Board of Directors, MedTech Europe, Brussels, Belgium; 3 Center for Global Health, Istituto Superiore di Sanità, Rome, Italy

**Keywords:** World Health Organization, prequalification, in vitro diagnostics, regulatory environment, low- and middle-income countries

## Abstract

In vitro diagnostic devices (IVDs) help clinicians determine specific conditions, monitor therapeutic efficacy, and prevent drug resistance development. While stringent regulatory authorities (SRAs) regulate IVDs in most high-income countries, regulatory authorities in many low- and middle-income countries (LMICs) are nonexistent or do not enforce rigorous standards. In 2010, the World Health Organization established its Prequalification of In Vitro Diagnostics (PQDx) program to ensure “access to safe, appropriate and affordable” IVDs, especially in LMICs with little or no domestic regulatory frameworks, thereby reaching underserved populations. However, challenges in PQDx policies and procedures include an overloaded pipeline, timelines not publicly available, confusion about which products PQDx focuses on, perceived burden for documenting changes to prequalified products, overlap with SRA approvals, and uncertainty around long-term financing. PQDx can maximize its impact by considering the perspective of IVD manufacturers; similarly, IVD manufacturers should exercise adequate quality control over their submissions and associated processes.

## Teaser

This viewpoint discusses challenges of the World Health Organization (WHO) Prequalification of In Vitro Diagnostics program, in particular its scope, processes, timelines, and financing. It is informed by manufacturers of in vitro diagnostic devices, with feedback from the WHO.

The United Nations (UN) Sustainable Development Goal 3 is to “ensure healthy lives and promote well-being for all at all ages” [1]. Achieving this goal requires quality-assured in vitro diagnostic devices (IVDs) that facilitate timely patient access to drug regimens (when clinically relevant), to treat patients, prevent onward transmission of infections, and monitor therapeutic responses (thereby reducing the risk of drug resistance emerging due to suboptimal treatment) [2]. IVDs must be reliable, robust (ie, functional in the intended settings of use, which sometimes include extreme operating conditions, such as dust or excessive temperature), and with acceptable sensitivity and specificity [3]. In low- and middle-income countries (LMICs), additional issues must be considered when choosing IVDs for use in national programs (eg, shortage of skilled laboratory personnel, unstable electricity supply, scarcity of laboratory equipment, and costs for equipment and consumables).

## Risks of Regulatory Control Absence

Stringent regulatory authorities (SRAs; including US Food and Drug Administration [FDA] and Conformité Européenne [CE] Marking) regulate IVDs in developed countries. Unfortunately, regulatory authorities in many LMICs are nonexistent or do not enforce rigorous standards for IVDs [2, 4]. This lack of regulatory control can lead to insufficient access to quality-assured and appropriate IVDs, resulting in the use of poor-quality tests potentially producing inaccurate or misleading results, inadequate monitoring of responses to therapy, and incorrect treatments, with potential consequences on the health of patients [4].

Antigen-detecting rapid diagnostic tests (RDTs) for malaria provide an example of the risks of using IVDs not properly evaluated. These RDTs have been available for >15 years, but quality assurance is still not universally enforced; nonvalidated RDTs with suboptimal performance are widely available despite little evidence of their functionality [4–6]. In response to this situation, companies that manufacture malaria RDTs were invited to submit products for the World Health Organization (WHO)/Foundation for Innovative New Diagnostics (FIND) malaria RDT evaluation program; since 2006, 247 products have been tested [7]. After December 2017, only antigen-detecting RDTs that meet WHO Prequalification of In Vitro Diagnostics (PQDx) requirements will be eligible for procurement by WHO [8]. Unfortunately, substandard malaria RDTs will remain available since a significant proportion are purchased through the private sector (and not procured by national programs). National regulatory authorities might benefit from WHO support to help address this issue.

## WHO Prequalification of In Vitro Diagnostics Program

WHO established its PQDx program to ensure “access to safe, appropriate and affordable [IVDs] of good quality,” especially in resource-limited settings with little or no domestic regulatory frameworks [9]. PQDx has been successful in achieving this and should be credited for establishing a process for IVDs that can be used by countries without well-established regulatory systems, reducing the risk of inappropriate IVDs being utilized in healthcare systems within resource-limited settings.

PQDx undertakes a comprehensive assessment of submitted IVDs through review of a product dossier, site inspections, and laboratory evaluation of products. PQDx site inspections are conducted according to the quality management standard ISO 13485:2003 [10], other appropriate international standards, and guidelines produced by the Global Harmonization Task Force on Medical Devices (GHTF) and the International Medical Device Regulators Forum (IMDRF, now replacing GHTF) [11, 12]. Once prequalified, reportable changes to IVDs and/or manufacturing sites must be submitted to WHO PQDx.

Prequalification was originally intended to guide eligibility for purchase by UN agencies; it is also used by non-UN donor agencies, procurement agents, and some LMICs as a procurement criterion. This criterion is sometimes inconsistently applied by purchasing agencies. Consequently, donors and government agencies may still purchase products made under less stringent quality systems [2, 5]. Moreover, certain IVDs are excluded from PQDx and evaluated by a different program within the WHO.

We consulted with IVD industry representatives involved in human immunodeficiency virus (HIV) and other disease areas to solicit their views of PQDx’s role and functioning, and their suggestions for improving the program’s contribution. This input, gathered from 14 companies and other independent experts, and further informed through discussions with PQDx, forms the basis for this viewpoint.

## DISCUSSION

### Prequalification of In Vitro Diagnostics Scope

PQDx accepts for assessment IVDs on the WHO procurement scheme: IVDs for the diagnosis and/or monitoring of HIV-1/2 and hepatitis C, IVDs for the diagnosis of malaria, point-of-care RDTs and/or technologies, products that are manufactured by the original product manufacturers (ie, the legal manufacturer), and product categories for which there are few other prequalified products [12]. WHO also encourages joint applications by original equipment manufacturers and companies purchasing finalized/semifinalized products and marketing these under their own brand names (ie, rebranding) [12]. As of 2018, PQDx’s scope will also include IVDs for the detection of *Vibrio cholerae* [13].

Periodically, consultations are carried out to determine if eligibility criteria should be updated in response to changing global health needs, WHO member states’ requirements and emerging relevant technologies. More transparency is needed on the rationale and process by which PQDx, through inputs received from member states, procurement agencies, donors, and WHO disease-specific programs, accepts diseases and technologies for assessment as this has a significant effect on product research, development, and production by interested parties (ie, academics, not-for-profit organizations, and industry, referred to below as IVD manufacturers).

WHO assessment mechanisms across various diseases are incoherent: some products are assessed through PQDx, others through different WHO programs (eg, tuberculosis [TB] IVDs assessed through the WHO Global TB Programme). There must be improved clarity about which disease states and associated products PQDx focuses on. A uniform WHO assessment mechanism for areas that lack access to quality-assured IVDs would be beneficial and PQDx should centralize oversight work for IVDs in all diseases covered by WHO. However, this should take place after streamlining the program to avoid overburdening PQDx staff and resources.

### Prequalification of In Vitro Diagnostics Processes

Changes to the product (eg, manufacturing site, components, and reagents) may trigger an assessment by PQDx, but manufacturers often find it challenging to determine whether a review is necessary. An unwanted consequence of the perceived lack of clarity in the guidelines is the belief that changes will prompt a resubmission of the entire dossier, with inevitable delays in approval and risks for continued procurement. In practice, however, PQDx has not requested full resubmissions; the perception may lie in the way PQDx communicates its processes. Moreover, the change notification process for PQDx is perceived as burdensome, although requirements are similar to those of other SRAs (FDA, Health Canada, the European Commission Notified Body Operations Group, and Singapore’s Health Sciences Authority). There exists no international overarching guidance around change procedures; global standards could benefit the regulatory community and the IMDRF would be well positioned to coordinate their development. New guidelines on reportable changes to prequalified IVDs were released in 2016; these may attenuate this perception [14]. Improving communication with IVD manufacturers may also advance mutual understanding.

Moreover, with uncertainty about the required information, lengthy back and forth communication with PQDx (with delays in response of a few weeks up to several months) can prolong timelines and increase the workload for both manufacturers and PQDx. It is acknowledged that the pipeline of products under review by PQDx is overloaded (too many products under review for the available capacity). Increasing the scope of the program’s work, including postmarketing surveillance, will only exacerbate this situation unless there is a considerable increase in staffing (and funding) or a reduction in other tasks. One option is to limit PQDx’s activities to only assessing the suitability of products for use in LMICs. It may also be more useful if PQDx focuses on IVDs that have not undergone SRA review at all. Streamlining the number and locations of the evaluations required, and the prequalification review process, would also increase the program’s efficiency.

### Duration and Transparency of Timelines

Manufacturers must adhere to strict timelines that are protracted and poorly defined, whereas PQDx is not bound by the same constraints. In addition, manufacturers are not fully aware of assessment timelines, planned and effective, which are not public and result in unpredictability. While several manufacturers need timeline extensions to complete the assessment process, others have experienced unnecessarily long timelines, potentially causing an inability to make commercial decisions, such as proactively ramping up manufacturing capacity and marketing of the product.

Any meaningful assessment process requires time, and considerations beyond time of market entry (ie, safety, quality, and performance) are most important. However, a lengthy prequalification process can have both healthcare and commercial implications. Companies need to achieve a return on their investment, and small companies are particularly vulnerable to lengthy financial pressures. Waiting too long for prequalification has led to a lack of sales and inadequate cash flow; this may have resulted in IVDs being withdrawn from the market or entering the market late, yielding negative consequences for populations most in need of timely access to quality IVDs. Delayed products can also become redundant due to emerging technologies, evolution of WHO guidelines, and/or changes in healthcare practice.

There have been efforts recently to shorten timelines and avoid past situations where timelines have been excessively long for some product categories. However, without transparency on effective timelines, this progress is difficult to evaluate. PQDx already shares with its donors detailed information on their timelines and should consider making some of this information public.

### Overlap With Other Regulatory Processes

Despite PQDx taking part in the IMDRF as an observer, some level of overlap and duplication occurs between the reviews conducted by PQDx and SRAs. In countries where premarket approval and, particularly, in-country evaluations exist, they may overlap considerably with PQDx, particularly in the independent performance evaluation component. On-site inspections by PQDx and country-specific clinical performance studies in each potential market after an IVD has already been evaluated by an SRA duplicate previous work, increase the manufacturer’s costs, and can delay access to and affordability of critically needed IVDs for several years [2, 5]. IVD manufacturers would prefer that SRA-approved products undergo PQDx processes that are more focused and shorter than the current abbreviated procedure [15]. There is an opportunity for PQDx to lead harmonization efforts in this area. The abbreviated process, which does not require submission of a product dossier but still relies on a short, focused inspection, may help but may still be redundant if evaluations and site inspections are requested while they have already been done for SRA approval. The Medical Device Single Audit Program (initiated by the IMDRF), which PQDx is a part of, may help alleviate this issue. PQDx should take advantage of this program rather than doing its own site inspections.

It should be noted, however, that different SRAs assign different risk categories to different diseases. HIV assays (including viral load, but not CD4 counts) are in the highest risk category for CE Marking; these must meet common technical specification criteria and undergo a more stringent review (including ongoing lot testing by reference laboratories in Europe) than devices used in other disease areas, where there is minimal review or where manufacturers can “self-declare” an assay to affix a CE Mark. Nevertheless, there have been products bearing the CE Mark that have been listed under PQDx notices of concern. The stringency of the CE Marking system should be strengthened in the coming years once new regulations [16] are fully implemented (following a transition period of 5 years). In preparation for this, the stringency of notified bodies has started to improve.

While new oversight processes are emerging, often with the goal of improving coordination, some of these are able to access the same funding sources that support PQDx; there are concerns that this could fragment efforts. Selected IVDs, which have a high public health impact but have not yet gone through or are undergoing PQDx or SRA review, can be reviewed by the UNITAID-supported Expert Review Panel for Diagnostics (ERPD) in response to a twice-yearly call by the Global Fund [17]. This mechanism is intended to allow products to be marketed while they are undergoing prequalification; procurement is time limited (the product must have been WHO prequalified within a year) [18]. In reality, countries often do not accept ERPD alone and rely on PQDx.

A common agreement on which authorities are responsible for which geographic and therapeutic areas would be very helpful for IVD manufacturers. This may be a way to reconcile the apparent difference in scope of most IMDRF members with those of PQDx, which focuses on aspects specific to LMICs’ often challenging operating conditions. Clarification of the different regulatory organizations’ requirements will assist manufacturers establishing realistic timelines.

### Financing of Prequalification of In Vitro Diagnostics

UNITAID funds most of PQDx [19], and manufacturers submitting products for prequalification pay nonrefundable fees to cover some costs [12]. It was proposed that manufacturers could contribute a proportion of their sales income to support PQDx. However, the impartiality of PQDx might be questioned if the program’s funding depends on IVD sales volumes. It might be assumed that “best-seller” IVDs would be fast-tracked ahead of essential but low-volume IVDs or that well-resourced manufacturers would be preferred because they can generate higher sales volumes than smaller companies. Manufacturers are also reluctant to provide funding on this basis because of market confidentiality issues. The majority of them are, however, willing to pay a slightly increased prequalification submission fee. Although the new financing model for both the WHO Prequalification of Medicines and Vaccines programs have been launched in early 2017, there is still no visibility on the new approach for PQDx.

### Beyond In Vitro Diagnostic Devices

Manufacturers seeking WHO Prequalification for male circumcision devices have faced similar issues (around communication, timelines, and processes). In some cases, no specific guidance exists for these medical devices (as in other regulatory bodies) and WHO has instead used IVD-specific processes for circumcision devices (eg, change procedure). This has led to confusion, with male circumcision device manufacturers trying to interpret guidance developed for IVDs.

### Recent Progress by Prequalification of In Vitro Diagnostics

In 2015, WHO surveyed IVD manufacturers to determine the impact of efforts to improve processes, timelines, and transparency [20]. The technical competence and attitudes of the WHO staff and external experts were described as strengths of the prequalification process. Yet, manufacturers requested improved support, better communication via the website and direct contacts, as well as shorter/more predictable and transparent timelines.

In a commendable effort, PQDx has started outsourcing evaluations to expert laboratories and restricting its involvement to issuance of standards and convening of consultations to discuss evaluation results for approval. Performance evaluations by WHO Prequalification Evaluating Laboratories can now be commissioned by manufacturers directly or through WHO [20]. Importantly, this performance evaluation step now takes place earlier in the assessment process while the dossier is still being screened for completeness (instead of conducting the evaluations only after completing dossier review). These changes are welcome and expected to reduce the prequalification timeline.

IVD manufacturers have requested more clarity about the requirements for dossier contents, and PQDx has issued useful documentation [12]. In Q3 2016, PQDx announced the development of a technical specifications series (TSS) to provide detailed dossier requirements for the prequalification of IVDs [21]. These documents align with best international practice and respond to the needs of WHO member states, especially resource-limited settings. Three TSS documents have been finalized following reception of feedback from industry and other stakeholders.

Regarding communication, this has considerably been improved recently (eg, with regular newsletters). Also, some harmonization across the WHO is taking place, for example, with the determinant of procurement eligibility for malaria RDTs moving from the WHO Global Malaria Programme (WHO Malaria RDT Product Testing Programme) to PQDx by the end of 2017 [22]. However, for this to represent an improvement, key technical expertise from the WHO Global Malaria Programme must not be lost, calling for close collaboration between the 2 separate entities within WHO; this is expected for other areas—including HIV and hepatitis—as well.

### In Vitro Diagnostic Device Manufacturer Responsibilities

It is important to note that IVD manufacturers also have responsibilities that contribute in a significant way to the efficiency and effectiveness of PQDx. Dossiers must be well organized, clearly written in English, and conform to PQDx instructions so that the assessor can locate information easily. Manufacturers must be completely transparent about regulatory versions of their tests so that PQDx understands precisely what product it is assessing. Information in package inserts must exactly match the information in the dossier. Truthfulness is essential; data integrity issues lead to a loss of trust that is very difficult to overcome. These issues are under the umbrella of the product dossier quality, well within the control of the manufacturer. It is understood that falling short on any of these issues can significantly lengthen the time to prequalification.

## CONCLUSIONS

PQDx is a valuable initiative that improves access to quality IVDs for underserved populations. Several parties, including United Nations agencies, WHO, the Global Fund, donors, national governments, IVD manufacturers (and the IVD industry in general) and, most importantly, patients have a mutual interest in the program functioning well. To achieve this, PQDx’s scope should be defined more coherently (both within WHO and with regards to SRAs) and timelines should be clarified and communicated publicly. Sustainable funding of PQDx, matched with a better-focused scope of work, is essential. Improving these aspects of PQDx should lead to enhanced access to quality-assured IVDs and better patient health, as well as better predictability for IVD manufacturers, which should accelerate the development process, increase commercial efficiency, and improve the return on investment.

The challenges with PQDx outlined in this viewpoint should be considered very carefully as they could become a deterrent for manufacturers wanting to submit dossiers for PQDx review, particularly as FDA approval and CE Marking are often sufficient for their business needs. For example, the Global Fund accepts CE Marking and FDA approval for IVDs having gone through the most stringent processes: Annex II List A and class III IVDs, respectively.

PQDx can only be sustainable if it understands the perspective of IVD manufacturers, focuses on quality gaps, and avoids duplication of existing stringent regulatory reviews. Similarly, IVD manufacturers must exercise adequate quality control over their submissions and associated processes. Strengthening the dialogue between PQDx and IVD manufacturers will promote collaboration and ensure everyone works together to facilitate the long-term, sustainable functioning of WHO PQDx.

## Notes

***Acknowledgments.*** The International AIDS Society’s Industry Liaison Forum thanks the IVD manufacturers and individuals surveyed for this project. World Health Organization (WHO) PQDx is also thanked for constructive feedback and insights, which have contributed to improving this commentary.

***Financial support.*** This work was supported by the International AIDS Society’s Industry Liaison Forum, through contributions from its 2016 and 2017 Gold Partners (Gilead Sciences, MSD, and ViiV Healthcare), Silver Partners (AbbVie, Alere, and Janssen), and Bronze Partners (Abbott, Beckman Coulter, bioLytical Laboratories, Cepheid, Cipla, Female Health Company, Lupin Pharmaceuticals, Omega Diagnostics, Roche Molecular Systems, and Sysmex Corporation). The International AIDS Society’s Industry Liaison Forum provided funding for Dr Wendy Smith, Wordsmiths International Ltd, to provide medical writing support for the initial draft of this commentary.

***Potential conflicts of interest.*** P. J. is President, Emerging Markets for diagnostics manufacturer Cepheid and is a member of the International AIDS Society’s Industry Liaison Forum Advisory Group. S. V. is a member of the International AIDS Society Governing Council. All other authors report no potential conflicts. All authors have submitted the ICMJE Form for Disclosure of Potential Conflicts of Interest. Conflicts that the editors consider relevant to the content of the manuscript have been disclosed.
